# Automated Electronic Health Record Data Extraction and Curation Using ExtractEHR

**DOI:** 10.1200/CCI.24.00100

**Published:** 2024-11-25

**Authors:** Tamara P. Miller, Kelly D. Getz, Edward Krause, Yun Gun Jo, Sandhya Charapala, M. Monica Gramatages, Karen Rabin, Michael E. Scheurer, Jennifer J. Wilkes, Brian T. Fisher, Richard Aplenc

**Affiliations:** ^1^ Aflac Cancer and Blood Disorders Center, Children's Healthcare of Atlanta, Atlanta, GA; ^2^ Department of Pediatrics, Emory University School of Medicine, Atlanta, GA; ^3^ Department of Biostatistics, Epidemiology, and Informatics, Perelman School of Medicine, University of Pennsylvania School of Medicine, Philadelphia, PA; ^4^ Department of Biomedical and Health Informatics, The Children's Hospital of Philadelphia, Philadelphia, PA; ^5^ Division of Hematology-Oncology, Department of Pediatrics, Baylor College of Medicine, Houston, TX; ^6^ Texas Children's Cancer and Hematology Centers, Texas Children's Hospital, Houston, TX; ^7^ Division of Pediatric Hematology/Oncology, Department of Pediatrics, University of Washington School of Medicine, Seattle, WA; ^8^ Division of Infectious Diseases, The Children's Hospital of Philadelphia, Philadelphia, PA; ^9^ Division of Oncology, The Children's Hospital of Philadelphia, Philadelphia, PA; ^10^ Perelman School of Medicine, University of Pennsylvania School of Medicine, Philadelphia, PA

## Abstract

**PURPOSE:**

Although the potential transformative effect of electronic health record (EHR) data on clinical research in adult patient populations has been very extensively discussed, the effect on pediatric oncology research has been limited. Multiple factors contribute to this more limited effect, including the paucity of pediatric cancer cases in commercial EHR-derived cancer data sets and phenotypic case identification challenges in pediatric federated EHR data.

**METHODS:**

The ExtractEHR software package was initially developed as a tool to improve clinical trial adverse event reporting but has expanded its use cases to include the development of multisite EHR data sets and the support of cancer cohorts. ExtractEHR enables customized, automated data extraction from the EHR that, when implemented across multiple hospitals, can create pediatric cancer EHR data sets to address a very wide range of research questions in pediatric oncology. After ExtractEHR data acquisition, EHR data can be cleaned and graded using CleanEHR and GradeEHR, companion software packages.

**RESULTS:**

ExtractEHR has been installed at four leading pediatric institutions: Children's Healthcare of Atlanta, Children's Hospital of Philadelphia, Texas Children's Hospital, and Seattle Children's Hospital.

**CONCLUSION:**

ExtractEHR has supported multiple use cases, including five clinical epidemiology studies, multicenter clinical trials, and cancer cohort assembly. Work is ongoing to develop Fast Health care Interoperability Resources ExtractEHR and implement other sustainability and scalability enhancements.

## Teaser

ExtractEHR is a low-cost software package to extract and curate electronic health record data in multiple use cases.

## INTRODUCTION

Many publications have described both the potential and limitations of the electronic health record (EHR) in facilitating clinical research.^1-4^ This transformative potential arises from the extensive, digitally accessible data describing patients' medical experience with previously unavailable granularity. The well-described limitations stem in part from the typically siloed data structures that hinder broad data access.^5^ Many investigators and commercial entities seek to break down these silos to create data sets that span institutions and enable practice-changing data analyses.

**CONTEXT**

**Key Objective**
To describe development and use cases of ExtractEHR, a software package that was developed to extract and process data from the electronic health record (EHR).
**Knowledge Generated**
The process of implementing and using ExtractEHR for customized, automated data extraction from the EHR across multiple hospitals is described. Use cases demonstrate the range of opportunities for which ExtractEHR can be beneficial.
**Relevance**
A software tool that can be used to extract data from EHR (here used in pediatric oncology)—obviously very useful.

Typically these efforts focus on adult health conditions with large numbers of patients and potential revenue for commercial entities. The Flatiron Health database is perhaps best known and has been used for multiple studies in cancer cohorts.^6-9^ Although extensive, Flatiron does not currently include sufficient numbers of pediatric cancer patients, and access to the data requires Flatiron approval. The Center for International Blood and Marrow Transplant (CIBMTR) is piloting an automated Data Transformation Initiative to extract EHR data, including demographics, diagnoses and dates, labs, therapies, responses, relapses, and transplant data for pediatric and adult patients across sites receiving stem-cell and cellular therapies. However, this is specific to CIBMTR forms and limited to patients receiving hematopoietic stem-cell transplants, and thus may not be generalizable to broader populations. Finally, REDCap Clinical Data Interoperability Services (CDIS) enables transfer of EHR data directly into REDCap for research purposes.^10^ Although user-friendly, the CDIS does not currently include radiology results or clinician notes, does not incorporate data cleaning or synthesis procedures, and does not directly support federating EHR data across institutions.^11^

In pediatrics, EHR data federation has been led by PEDSNet, which federates data from multiple free-standing pediatric institutions and has supported multiple clinical research studies.^12^ This work has included creation of a computable phenotype identifying patients with acute leukemia and lymphoma.^13^ However, this computed phenotype does not distinguish between acute leukemia subtypes, an important potential limitation. Other pediatric groups have developed algorithms to identify local patient cohorts and to extract targeted critical clinical data, such as the cumulative anthracycline exposure.^14^ However, these efforts have been limited to single institutions and do not generate the larger scale, multi-institutional data sets needed to address complex clinical epidemiology questions.

In response to the need for a comprehensive data set inclusive of patients from multiple hospitals that can be used to answer clinical pediatric oncology research questions, we developed a software tool called ExtractEHR. We aim to describe the procedures used to develop and implement ExtractEHR, provide use cases across a range of clinical research applications, and discuss future potential applications.

## METHODS

### Overview

**FIG 1.**
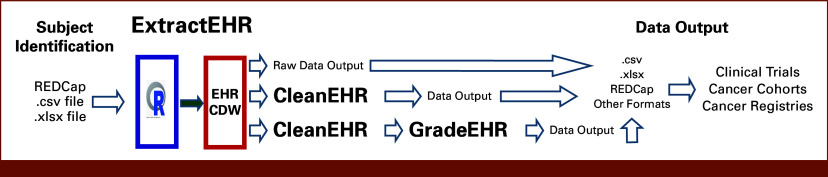
EHR processing toolkit. EHR, electronic health record.

**FIG 2.**
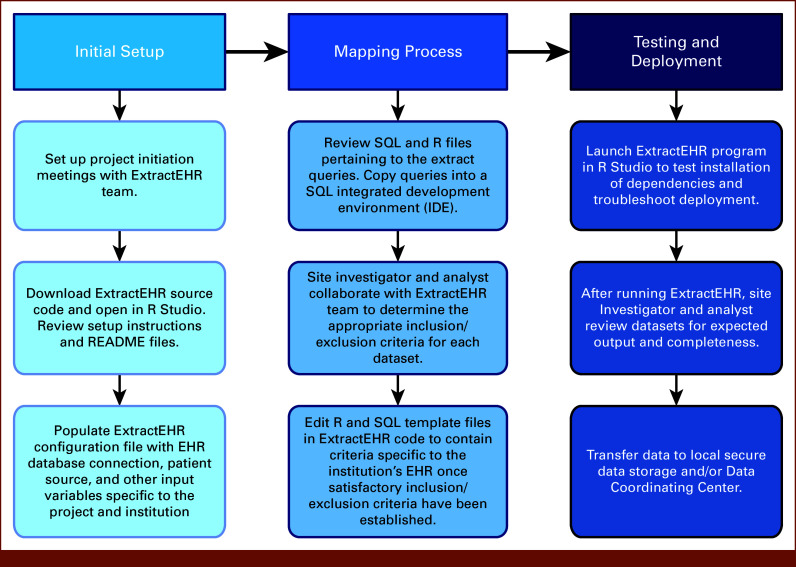
ExtractEHR setup. EHR, electronic health record; SQL, Structured Query Language.

ExtractEHR is a software package written in R that extracts various structured, semi-structured, and unstructured data components from EHR systems. Data components include discrete chart elements, such as laboratory results or flowsheet data, and free-text documents, such as clinician notes or radiology reports. The specific components used for a given project are dictated by the project's protocol. During implementation, ExtractEHR is programmed to extract data from the EHR database for the patients and boundary dates needed for the specific use case. In addition to comprehensive EHR data extraction, we have developed two modules for postextraction EHR data cleaning and processing. CleanEHR cleans raw data for downstream analytic use. GradeEHR computes grades per Common Terminology Criteria for Adverse Events (CTCAE) for cleaned laboratory results and other data analyses. Figure 1 provides a schematic representation of the ExtractEHR, CleanEHR, and GradeEHR inputs, outputs, and processes, whereas Figure 2 provides an overview of ExtractEHR onboarding and setup.

### Initial Setup

Implementation of ExtractEHR begins with an installation agreement between the site and the ExtractEHR Team. ExtractEHR is free but is not open-source due to the proprietary nature of the connections to the EHR vendors. ExtractEHR code is stored in a private GitHub repository with access given during initial implementation. ExtractEHR setup requires users to populate a configuration file with credentials for the source system connections and other settings that guide the data extraction process. Each institution must also complete a localized mapping process, in which specific data are defined and added to the template Structured Query Language (SQL) files included with the tool. Although institutions using the same brand of EHR system share a common data model, the data labels assigned to data elements used by ExtractEHR may differ between sites. Mapping ensures that data elements will be properly extracted and has been accomplished at Epic, Cerner, and All Scripts sites.

Mapping can be comprehensive across a data element type (eg, all laboratory tests) or tailored to include targeted elements (specific laboratory tests for a given use case). Pan-extraction (eg, all laboratory tests) substantially minimizes the mapping work required at ExtractEHR setup. However, it shifts the identification of individual components downstream in the analytic processing pipeline. In either mapping strategy, ExtractEHR may be used repeatedly for different use cases at the institution, unless other EHR components are desired or the EHR system undergoes a significant update with changes to the underlying data structure. Table 1 presents extracted data categories.

**TABLE 1.** Data Element Categories Captured by ExtractEHR

Data Element Categories	Type of Data	LEARN	AdOPT	Cooperative Group Trials	SEER	CCSS
Patient demographics	Structured	X			X	X
Encounters: Inpatient, day hospital, emergency room, and outpatient	Structured	X			X	X
Flowsheet data (vital signs)	Structured	X			X—only height and weight	
Medications ordered, including outpatient prescription orders	Structured	X			X	X
Medications administered	Structured	X			X	X
Laboratory test results, including microbiology test results	Structured	X	X	X	X	
Radiology result reports	Semi-structured	X			X	X
Pathology result reports	Semi-structured	X			X	X
Procedures	Structured	X			X	X
Clinician notes	Unstructured	X			X—only includes oncology clinician notes	X
Genomic data, if available	Variable by site	X			X	

Abbreviations: AdOPT, Outcomes of Human Adenovirus (HAdV) Infection and Disease in Pediatric Allogeneic Hematopoietic Cell Transplant Recipients: A Multicenter Observational Cohort; CCSS, Childhood Cancer Survivorship Study; EHR, electronic health record; LEARN, Leukemia Electronic Abstraction of Records Network; SEER, Surveillance, Epidemiology, and End Results; Trials, Cooperative Oncology Group Clinical Trials.

### Operation and Output

**FIG 3.**
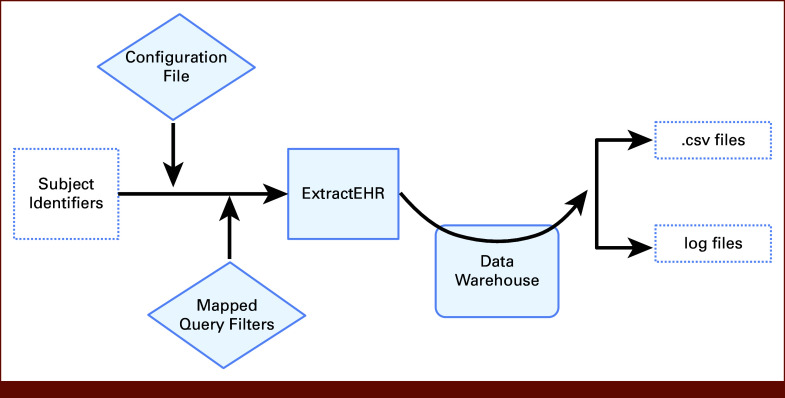
ExtractEHR process overview. EHR, electronic health record.

**FIG 4.**
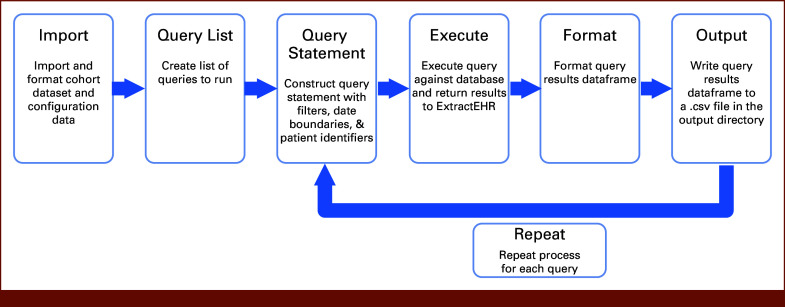
ExtractEHR query process overview. EHR, electronic health record.

Figures 3 and 4 display the process for operating ExtractEHR. Once the ExtractEHR initial setup is completed and the patients (identified by medical record number [MRN] or patient name and date of birth) and date boundaries (eg, date of birth or start/end dates of therapy courses) are identified, ExtractEHR extracts the data elements for that project. When the program is launched, ExtractEHR first loads the configuration file and establishes a connection to the EHR database. With the initial settings in place, the patient identifiers and data boundaries are loaded into a tabular data object called a dataframe and then formatted for insertion into SQL queries. For each component of the extraction, the tool loads a query template and, using SQL parametrization, inserts the patient IDs, filter criteria, and date boundaries to create a complete query statement. The query is transmitted to the database server, executed against the EHR database, and the results are transmitted back to ExtractEHR and stored in a dataframe. The resulting dataframe is then formatted for output, which differs on the basis of the EHR component and may include unit conversions, organization of free-text notes and comments fields, and trimming records on the basis of individual patient date boundaries. Depending on the use case, the ExtractEHR code can also remove MRNs and replace them with study IDs. For example, for a clinical trial using ExtractEHR to provide data, the MRNs would be removed and trial study ID would be appended. The formatted dataframe is then exported to a CSV file located in the output directory included with the tool or may be imported into REDCap, and the process can be repeated for additional queries.

## POSTEXTRACTION PROCESSING

We have developed two postextraction modules for data cleaning and processing. Using a series of rules defined by the ExtractEHR clinical team, CleanEHR removes false or duplicate results from the extracted data set, standardizes data element formatting, and creates pre- and postcleaning summary metrics of each file for quality control purposes.^15^ Similar to ExtractEHR, CleanEHR requires a configuration file, which contains variables for site and project names, filepaths, and optional database connection parameters, allowing cleaned data sets to be written to CSV or uploaded into a database. CleanEHR also includes a set of file cleaning parameters, which define additional settings, such as data processing workflows and exclusion values. CleanEHR identifies suspected false positives, such as electrolyte or hematologic laboratory results that change from being abnormal to normal in less than an hour.^15^ False-positive results from improper specimen collection or handling, such as a hemolyzed potassium result (identified in the comment field for the laboratory result), are removed.^15^ Cleaning metric reports are generated, which include raw frequency distributions (including procedure names, component names, sources, and reference units), lab result values, missing results, references values, summary statistics, and cleaned frequency distributions.

The second postextraction module is GradeEHR that grades each laboratory result per CTCAE v5 definitions. Like CleanEHR, GradeEHR runs centrally on data sets produced by CleanEHR. GradeEHR requires a configuration file (providing the site name, filepaths, and optional source and target database connection information), as well as a set of grading parameters that define the variable names, ranges, and grades, and the CleanEHR output data set. For each laboratory AE, GradeEHR loads the appropriate laboratory result data set, assigns a CTCAE grade to each record, and calculates whether the AE has normalized within a specified time interval. The graded data sets can be exported to CSV or loaded into a database. Currently, GradeEHR is capable of grading 25 laboratory AEs, with plans for additional laboratory AEs to be added. GradeEHR also generates a grading metrics report, which includes time difference frequency distributions, AE grade frequency distributions, and cross-tabulations of AE grades.

## ACCURACY

The accuracy of the ExtractEHR and postextraction CleanEHR and GradeEHR modules was tested in pediatric patients with AML at a single site.^15^ ExtractEHR had a sensitivity of over 98% for all laboratory AEs, whereas manual AE reports ascertained by clinical research associates had a sensitivity of 0%-21.1%.^15^ AE reporting errors included false-negative and false-positive results and incorrect grading, both ameliorated by the software packages.^15^

## POSTEXTRACTION DATA TRANSFER

Data management of extracted but unprocessed data is determined by use case requirements. Site data may be transferred directly to a third party or a central data coordinating center (DCC). In all use cases, site-specific extracted data are stored locally at each site in the extracted format. For example, laboratory data obtained by mapped data queries are stored as individual specific laboratory data files. Laboratory data obtained from pan-extracted, unmapped processes are stored in a single file containing multiple laboratory results within the file. Currently, CleanEHR and GradeEHR are only run at the DCC. Thus, use cases requiring cleaned or graded data require unprocessed data to be at least temporarily resident at the DCC. Cleaned and graded data may be retained centrally or passed on to a third-party recipient. Site-specific cleaned and graded laboratory data are always provided to the individual site.

## RESULTS

### Leukemia Electronic Abstraction of Records Network

ExtractEHR has been implemented at four large pediatric hospitals that comprise the Leukemia Electronic Abstraction of Records Network (LEARN) collaborative network. The LEARN cohort includes patients age 0-21 years treated for de novo AML or acute lymphoblastic leukemia (ALL) who received at least one complete course of chemotherapy at one of the included sites. LEARN currently includes Children's Healthcare of Atlanta (CHOA), Children's Hospital of Philadelphia (CHOP), Texas Children's Hospital, and Seattle Children's Hospital with several other sites being onboarded. Institutional Review Board (IRB) approval for LEARN research was obtained at all sites with waiver of informed consent.

LEARN has produced five peer-reviewed publications to date, demonstrating the utility of collecting granular data from the EHR for answering clinical research questions.^16-20^ A recent *Lancet Haematology* publication described rates of laboratory AEs in children with ALL and AML and delineated that commonly occurring laboratory AEs occurred more frequently when identified using ExtractEHR than had previously been reported in the data collected on Children's Oncology Group (COG) clinical trials.^17^ Strikingly, the most common laboratory abnormalities in both ALL and AML had log-fold differences in frequency between data identified in the ExtractEHR-enabled LEARN cohort and manually reported COG trial data. Additionally, the ExtractEHR-enabled LEARN data included patients regardless of clinical trial enrollment and thus constitute comprehensive, real-world data. In a second publication, ExtractEHR-ascertained data were used to examine treatment-associated hepatotoxicity in a diverse sample of children with ALL.^20^ Other recent laboratory data–based publications include the description of lymphocyte count recovery after AML chemotherapy, acute kidney injury after ALL and AML chemotherapy, and differences in severity of illness at presentation across racial/ethnic groups.^16,18,19^

ExtractEHR can also ascertain complex phenotypes that may be poorly defined by discrete data EHR data elements, that is, diagnosis codes. For example, ExtractEHR-ascertained data have been used to identify typhlitis, a serious and potentially life-threatening complication in leukemia treatment, in a LEARN subcohort.^21^ This paper reported an automated algorithm combining discrete EHR elements and natural language–processed free-text clinician notes to identify typhlitis. The algorithm outperformed manual abstraction with a higher sensitivity and a log-fold reduction in the number of chemotherapy courses requiring manual review.^22^

### Outcomes of Human Adenovirus Infection and Disease in Pediatric Allogeneic Hematopoietic Cell Transplant Recipients: A Multicenter Observational Cohort (AdOPT)

AdOPT is a mixed prospective and retrospective multicenter observational cohort study investigating Human Adenovirus infection and disease in pediatric allogeneic stem-cell transplant recipients. ExtractEHR is used at three of the 10 participating sites to obtain laboratory data; the other seven sites abstract laboratory data manually. The raw laboratory data obtained via ExtractEHR are transferred to the central data analysis team for import into the study's REDCap database. Sites using ExtractEHR report shorter chart abstraction times than those using manual laboratory data collection. Since the study population includes patients receiving an allogeneic stem-cell transplant for various conditions, the AdOPT study demonstrates the broad applicability of ExtractEHR. IRB approval for the AdOPT study was obtained at all sites with informed consent obtained for study participants.

### Cooperative Oncology Group Clinical Trials

In the current COG Phase III clinical trial for high-risk neuroblastoma, ANBL1531 (ClinicalTrials.gov identifier: NCT03126916), ExtractEHR is being used at CHOP and CHOA to identify and grade laboratory AEs. This study subaim will identify the highest grade of specified laboratory AEs by reporting period and assess the number of AEs detected and the amount of time required for each AE to be reported both before and after ExtractEHR implementation. In addition, EHR ascertainment of laboratory test results is being evaluated on a joint trial between the COG Pediatric Early Phase Clinical Trials Network and the Pediatric Brain Tumor Consortium.^23,24^ In the recently completed study (ClinicalTrials.gov identifier: NCT05020951), ExtractEHR was used to extract and electronically transfer raw laboratory data from five participating sites to the cloud-based Medidata Rave Electronic Data Capture system.^25^ IRB approval and informed consent were obtained at all sites participating in ANBL1651. IRB approval with waiver of informed consent was obtained at one site for the NCT05020951 trial with IRB determination at all other sites that the trial was not human subjects research.

### SEER Cancer Registry Pilot

The Surveillance, Epidemiology, and End Results (SEER) program of the National Cancer Institute includes 22 registries with collection of patient demographics, cancer information, and mortality and survival data.^26^ Cancer registries obtain data through provider reporting, which is required by law, but is labor-intensive and can be incomplete, inaccurate, and inconsistent.^27^ An ongoing pilot project has used ExtractEHR to ascertain and transfer demographics, clinical notes, laboratory results, medications ordered and administered, pathology reports, procedures, radiology reports, visits (clinic and inpatient), and vital signs (height and weight) from CHOA to the Georgia SEER Registry. Additional pilot testing in the Washington State, Texas, and New York SEER registries was recently completed. SEER pilot data are also shared with the National Childhood Cancer Registry (NCCR). IRB approval and consent were not obtained as this work was determined to be part of federally mandated cancer surveillance.

### Childhood Cancer Survivor Study

The Childhood Cancer Survivor Study (CCSS) contains data on approximately 30,000 childhood cancer survivors and has enabled many high-impact publications that have defined the care of childhood cancer survivors. CCSS relies on manual data abstraction of approximately 200 data elements, including chemotherapy exposure. ExtractEHR is being tested at five sites to evaluate the feasibility of automating capture of elements in the medical record abstraction form, particularly chemotherapy. The protocol supporting this work received IRB approval with waiver of informed consent.

## DISCUSSION

The successful implementation of ExtractEHR across a range of use cases demonstrates the broad potential applicability of ExtractEHR in pediatric cancer research, specifically the ability to facilitate development of real-world patient cohorts and to support clinical data registries. In these contexts, ExtractEHR may have particular value as a cost-effective tool for inclusive case identification regardless of clinical trial enrollment status. This capacity should address many of the well-described biases in research cohorts derived from clinical trials.^28^

As with any tool, ExtractEHR has important limitations that vary in impact across use cases. The primary limitation is the requirement for installation behind a local institutional firewall. Successful installation requires close collaboration between the ExtractEHR team and the local site team that typically comprises a clinical champion and a technical analyst. Support from institutional Information Systems is crucial, and the clinical champion's role in facilitating this support should not be underestimated. The requirement for local mapping, which can be labor-intensive, is another limitation. Although pan-extraction reduces local mapping effort, it requires additional downstream data processing before data analyses. In addition, depending on the number of patients included, pan-extraction may create very large data files. Finally, some institutions may require informed consent for certain EHR components, such as genomic data, and data sharing may require deidentification, which is challenging with unstructured EHR components. Work is ongoing to address this limitation by incorporating postextraction code to deidentify data elements or identify specific parts of each EHR component that do not include protected health information but retain clinical value. Use of existing packages such as Philter may also facilitate overcoming these challenges.^29^

Despite these limitations, ExtractEHR has several advantages. First, once installed, the package can be used for multiple, repeated use cases that can span varying, unrelated patient cohorts. These use cases can focus on any pediatric disease wherever the source population can be readily identified by a local site investigator. As an example, ExtractEHR has been used to extract data for children with chronic kidney disease at a single institution and is in process at other sites that are part of a multicenter collaboration. Second, once extracted, the data can be used not only for the defined use case but also for other local, secondary data analyses. This is particularly applicable when the mapped laboratory extraction approach is used, as that provides individual laboratory data files to the investigator. Furthermore, while ExtractEHR requires mapping at each institution, ExtractEHR does not require data transformation to a specific data format and rather extracts data as the institution has determined they should be stored. This provides an alternative to other existing data extraction methods that require use of a common data model or data standard. Finally, as an academically licensed software product, the only incurred costs are local site investigator and staff time.

As next steps, we are developing a Fast Health care Interoperability Resources (FHIR) version of ExtractEHR to determine whether local mapping requirements may be decreased or eliminated in specific use cases. The FHIR standard for information exchange simplifies EHR data sharing across institutions^30^ and has been widely adopted throughout the health care industry. The development of an FHIR ExtractEHR tool and broader ExtractEHR dissemination and use are crucial components of a sustainability strategy for ExtractEHR.
